# Accidental Intrathecal Injection of Tranexamic Acid

**DOI:** 10.1155/2012/646028

**Published:** 2012-03-26

**Authors:** Khaled Mahmoud, Amany Ammar

**Affiliations:** Faculty of Medicine, Minoufiya University, Shebin El Kom, Minoufiya 32511, Egypt

## Abstract

Tranexamic acid (TXA) is a popular antifibrinolytic drug that is commonly used in patients with bleeding disorder. Major morbidities and mortalities have been reported following inadvertent intrathecal injection of TXA. In this paper, inadvertent intrathecal injection of TXA has resulted from similarities in appearance between TXA and heavy bupivacaine 0.5% ampoules. The patient experienced severe pain in the back and gluteal region upon injection in association with systemic hypertension and tachycardia followed by generalized myoclonic seizures and ventricular fibrillation.

## 1. Introduction

The use of antifibrinolytic agents including tranexamic acid (TXA), aprotinin, and aminocaproic acid is popular in cardiac, gynecologic, and obstetric surgeries to overcome the increased fibrinolytic activity associated with these procedures [1]. Major complications in anesthesia practice have been reported from human errors like lack of vigilance, wrong labeling or presentation of the syringes and ampoules, or underestimation of the double-checking concept [2]. In this case report, TXA was injected intrathecally instead of hyperbaric bupivacaine 0.5%, because both ampoules were having the same appearance with consequent development of myoclonic seizures and ventricular fibrillation.

## 2. Case Description

Institutional review board approval and patient consent were obtained prior to reporting the case. A 54-year-old man was scheduled for lower-limb skin grafting. The patient was thoroughly examined and investigated and confirmed to be ASA I physical status. Spinal anesthesia was done with the patient in the sitting position at the L4-L5 interspace, using a 22-gauge needle. The anesthesiologist nurse prepared 3 mL of 0.5% hyperbaric bupivacaine for the anesthesiology resident. Upon injection of bupivacaine, the patient started complaining of severe pain in the back and gluteal region. The anesthesiologist attributed this pain to intraneural injection. No manifestations of sensory or motor block were noted, and the patient continued in severe intractable pain associated with elevation of the arterial blood pressure to 170/95 and pulse rate to 130. The patient received 100 *μ*g of fentanyl for analgesia and 2 mg of midazolam for sedation and put on oxygen face mask. Ten minutes later, the patient developed generalized myoclonic seizures. Consequently, the patient received propofol 200 mg and succinylcholine 80 mg for tracheal intubation and was mechanically ventilated and then transferred to the ICU. The attending anesthesiologist suspected accidental intrathecal injection of a wrong drug and discovered a used TXA ampoule (500 mg in 5 mL) in the trash can. In the ICU, the patient became feverish (40°C), and 2 gm IV acetaminophen was given. Three hours later, the patient got ventricular fibrillation that responded to electrical shock of 300 J. Subsequent treatment included propranolol, amiodarone, lidocaine, and mannitol in addition to continuous mechanical ventilation. Twenty-four hours later, the patient was weaned from mechanical ventilation, extubated, and fully recovered without any neurologic sequelae.

## 3. Discussion

 Tranexamic acid is a competitive inhibitor of plasminogen activation and a noncompetitive inhibitor of plasmin at higher concentrations. Its use in humans is generally well tolerated, and its complications are minimal and include mainly gastrointestinal upsets [1]. However, neurotoxicity and seizures have been reported in animal studies [3–5]. Furthermore, Yamaura and coworkers reported elevation of the systemic and intracranial pressure by direct cerebral application of TXA for treatment of ruptured intracranial aneurysm in animal models [4]. However, we know little about effect of TXA in case it is applied directly to the subarachnoid space in humans. In 1988, Wong and colleagues [6] reported accidental intrathecal injection of 75 mg TXA in adult patient undergoing appendectomy operation. The patient developed persistent sensory block of both lower extremities in addition to fever, myoclonus, and clonic convulsions that progressed to a generalized seizure that responded to intravenous diazepam, and the patient was fully recovered without any neurologic deficits. In 1999, De Leede-van der Maarl and coworkers [7] reported accidental intrathecal injection of 50 mg TXA in a 68-year-old man. The patient experienced refractory seizure that was treated by both diazepam and sodium thiopental. Furthermore, the patient complained of paresis in all extremities that resolved over time but with persistent bilateral peroneal palsy. In 2003, Yeh and colleagues [8] reported inadvertent intrathecal injection of 500 mg of TXA. The patient developed generalized convulsions and hypertensive response followed by refractory ventricular fibrillation that ended the life of the patient. The authors attributed this sequelae to a massive sympathetic discharge induced by TXA which was near to the events encountered in our case. Whereas triggering of seizures was explained by suppression of the inhibitory gamma-aminobutyric acid- (GABA)-A receptors in the cerebral cortex or lowering of cerebral blood flow with consequent cerebral ischemia [9, 10]. In 2007, Garcha and colleagues [11] reported mortality after accidental intrathecal injection of TXA. In 2011, Kaabachi et al. [12] reported accidental intrathecal injection of 80 mg TXA in a 30-year-old man. The patient experienced severe pain in the back and gluteal region and recurrent attacks of polymyoclonus and seizures, in addition to recurrent episodes of ventricular tachycardia that subsided spontaneously. The patient was fully recovered within 4 days without any neurologic sequelae.

## 4. Conclusion

We should emphasize that all the previously mentioned case reports have resulted from confusion between TXA and hyperbaric bupivacaine 0.5% ampoules that were having the same appearance. We recommend that critical drugs like the drugs used for spinal anesthesia have unique appearance and package so possibility of confusion is remote. At the same time, both the anesthesiologist and nurse must check the ampoule label precisely and stick to the double-checking concept.
